# *Barley yellow dwarf virus* Infection Leads to Higher Chemical Defense Signals and Lower Electrophysiological Reactions in Susceptible Compared to Tolerant Barley Genotypes

**DOI:** 10.3389/fpls.2018.00145

**Published:** 2018-03-06

**Authors:** Maria K. Paulmann, Grit Kunert, Matthias R. Zimmermann, Nina Theis, Anatoli Ludwig, Doreen Meichsner, Ralf Oelmüller, Jonathan Gershenzon, Antje Habekuss, Frank Ordon, Alexandra C. U. Furch, Torsten Will

**Affiliations:** ^1^Department of Plant Physiology, Matthias-Schleiden-Institute for Genetics, Bioinformatics and Molecular Botany, Faculty of Biological Sciences, Friedrich Schiller University Jena, Jena, Germany; ^2^Department of Biochemistry, Max Planck Institute for Chemical Ecology, Jena, Germany; ^3^Department of Biology, Elms College, Chicopee, MA, United States; ^4^Institute for Resistance Research and Stress Tolerance, Federal Research Centre for Cultivated Plants, Julius Kuehn-Institute, Quedlinburg, Germany

**Keywords:** *Barley yellow dwarf virus*, electrical penetration graph, electropotential waves, phloem, phytohormones, reactive oxygen species, sieve element, xylem

## Abstract

*Barley yellow dwarf virus* (BYDV) is a phloem limited virus that is persistently transmitted by aphids. Due to huge yield losses in agriculture, the virus is of high economic relevance. Since the control of the virus itself is not possible, tolerant barley genotypes are considered as the most effective approach to avoid yield losses. Although several genes and quantitative trait loci are known and used in barley breeding for virus tolerance, little is known about molecular and physiological backgrounds of this trait. Therefore, we compared the anatomy and early defense responses of a virus susceptible to those of a virus-tolerant cultivar. One of the very early defense responses is the transmission of electrophysiological reactions. Electrophysiological reactions to BYDV infection might differ between susceptible and tolerant cultivars, since BYDV causes disintegration of sieve elements in susceptible cultivars. The structure of vascular bundles, xylem vessels and sieve elements was examined using microscopy. All three were significantly decreased in size in infected susceptible plants where the virus causes disintegration of sieve elements. This could be associated with an uncontrolled ion exchange between the sieve-element lumen and apoplast. Further, a reduced electrophysiological isolation would negatively affect the propagation of electrophysiological reactions. To test the influence of BYDV infection on electrophysiological reactions, electropotential waves (EPWs) induced by leaf-tip burning were recorded using aphids as bioelectrodes. EPWs in infected susceptible plants disappeared already after 10 cm in contrast to those in healthy susceptible or infected tolerant or healthy tolerant plants. Another early plant defense reaction is an increase in reactive oxygen species (ROS). Using a fluorescent dye, we found a significant increase in ROS content in infected susceptible plants but not in infected tolerant plants. Similar results were found for the phytohormones abscisic acid and three jasmonates. Salicylic acid levels were generally higher after BYDV infection compared to uninfected plants. Heat stimulation caused an increase in jasmonates. By shedding light on the plant defense mechanisms against BYDV, this study, provides further knowledge for breeding virus tolerant plants.

## Introduction

The barley yellow dwarf disease caused by different viruses of the genus *Luteovirus* [e.g., *Barley yellow dwarf virus* (BYDV) *-PAV*] and the genus *Polerovirus* (e.g., *Cereal yellow dwarf virus-RPV*) of the family *Luteoviridae*, infects a wide range of plants including, e.g., maize, wheat, rye, oat and barley and causes one of the most serious viral diseases in cereal crops and grasses worldwide (D’Arcy, 1995). The primary symptoms of infected barley are stunted growth and yellow discoloration of leaves. The virus is phloem restricted and is transmitted in a persistent manner by many aphid species, e.g., *Rhopalosiphum padi* and *Sitobion avenae* (Slykhuis, 1967; Jensen, 1969). BYDV infection leads to a collapse of sieve elements accompanied by an accumulation of “wound gum” resulting in necrosis (Esau, 1957). Esau (1957) also showed that companion cells (CCs) and parenchyma cells are affected by necrosis as well. How this affects phloem physiology has not yet been studied.

The control of the aphid vector with insecticides is one approach to prevent BYDV infection. However, for environmental reasons and the risk of resistance development, the use of insecticides is being actively discouraged. Thus, growing virus tolerant cultivars may be one of the most suitable ways to reduce the negative impact of virus infection on agriculture. Susceptibility to a virus means that it can multiply and spread (move from cell to cell) inside its host plant and causes strong disease symptoms. A tolerant genotype is characterized by weak or no disease symptoms even though infection, multiplication and spread are the same as in susceptible genotypes (Cooper and Jones, 1983). One form of tolerance/resistance of BYDV in barley is mediated by the gene *Ryd2* (Schaller et al., 1964). The *Ryd2* based tolerance is used in, e.g., the winter barley cultivar ‘Vixen’ since 1986 (Parry and Habgood, 1986) but until now no information was available about the functional background of this tolerance. Other genes, such as *Ryd3* (Niks et al., 2004) and *Ryd4*^Hb^ (Scholz et al., 2009) and additional quantitative trait loci (Scheurer et al., 2001) are known as well. Although the modes of action of these genes are not known, it is assumed that these prevent the negative effects of the virus on the phloem.

The phloem is composed of sieve elements (SEs), CCs and phloem parenchyma cells (PPCs), which are mainly involved in long distance transport of nutrients (van Bel, 2003). SEs are elongated cells that contain only a plasma membrane, a few mitochondria, a parietally located smooth endoplasmic reticulum, SE plastids and an extensive set of phloem-specific proteins (Giavalisco et al., 2006). The perforated sieve plates, located at the terminal ends of SEs, are modified cell walls that allow the flow of sap from one SE to the next one. Mass flow inside sieve tubes is driven by an osmotic pressure gradient between source and sink tissues (Münch, 1930; Knoblauch et al., 2016) that distributes carbohydrates, amino acids, proteins, vitamins, phytohormones, and other signaling molecules throughout the whole plant.

Besides the transport of nutrients, the phloem is also involved in long-distance communication through electropotential waves (EPWs) (Furch et al., 2007, 2009). EPWs have been recorded in response to mechanical and physical stimuli, such as wounding, cold, heat, and electrical shocks (Fromm and Spanswick, 1993; Rhodes et al., 1996; Mancuso, 1999; Furch et al., 2007) but also in response to biotic stimuli such as feeding by caterpillars (Salvador-Recatalà et al., 2014; Zimmermann et al., 2016). EPWs include features of action potentials and variation potentials (Hafke et al., 2009) and are involved in signaling regarding growth regulation, adjustment of photosynthesis and respiration, and defense (Trêbacz et al., 2006). Burning induced EPWs are associated with distant occlusion of sieve tubes by proteins and callose deposition (Furch et al., 2007, 2009, 2010; Will et al., 2009).

Defense signaling against plant pathogens also has chemical components. During biotrophic pathogen attack salicylic acid (SA) and its derivative, methyl salicylate, are known to be key signals in systemic acquired resistance (SAR) and the hypersensitive response (HR) (Métraux et al., 1990; Vlot et al., 2009; Dempsey et al., 2011). The (+)-7-*iso*-jasmonoyl-_L_-isoleucine (JA-Ile) but also other jasmonic acids (JA) – amino acid conjugates like JA-valine (JA-Val), have been discussed as general inter- and intracellular signaling compounds typically involved in multiple defense reactions to both, necrotrophic microbial pathogens and herbivore attack (Wang et al., 2007; Verhage et al., 2011; Koo and Howe, 2012; Pel and Pieterse, 2013; Wasternack and Hause, 2013). Various abiotic stimuli lead to the overproduction of reactive oxygen species (ROS) in plants. ROS also control many processes like programmed cell death, abiotic stress responses, pathogen defense and systemic signaling (Gill and Tuteja, 2010).

To learn more about the defense responses of barley plants to BYDV infection, we investigated viral-induced changes in the structure of vascular bundles, which could alter mass flow and therefore the propagation of EPWs and chemical signals. In addition, we measured the propagation of EPWs in the sieve tubes and measured defense responses, including the accumulation of ROS and phytohormones.

## Materials and Methods

### Aphid Cultivation

*Sitobion avenae* and *R. padi* were reared on 14–28 day old plants of *Hordeum vulgare* cv. Rubina in a greenhouse under controlled-environment conditions at 20°C and a 16 h:8 h L:D regime. Aphids were maintained in perspex cages with large gauze-covered windows.

### Plant Material and General Experimental Set-ups

*Hordeum vulgare* cv. Rubina (BYDV-susceptible) and *H. vulgare* cv. Vixen (BYDV tolerant) plants were grown in a greenhouse at 20°C with natural lighting supported by artificial light to maintain a 14 h:10 h L:D period. Two to three week old plants of both cultivars were infested with (i) virus free or (ii) viruliferous (BYDV-PAV; Sip et al., 2006) aphids of the species *R. padi*. For both treatments five adult apterous aphids were placed in a clip cage on the first leaf of a single plant for a period of 48 h. Subsequently, aphids were removed mechanically and all plants were additionally sprayed with an insecticide (Confidor^®^ 0.035%; Bayer, Germany). Treated plants were cultivated in a climate chamber under the same conditions as in the greenhouse.

**FIGURE 1:**
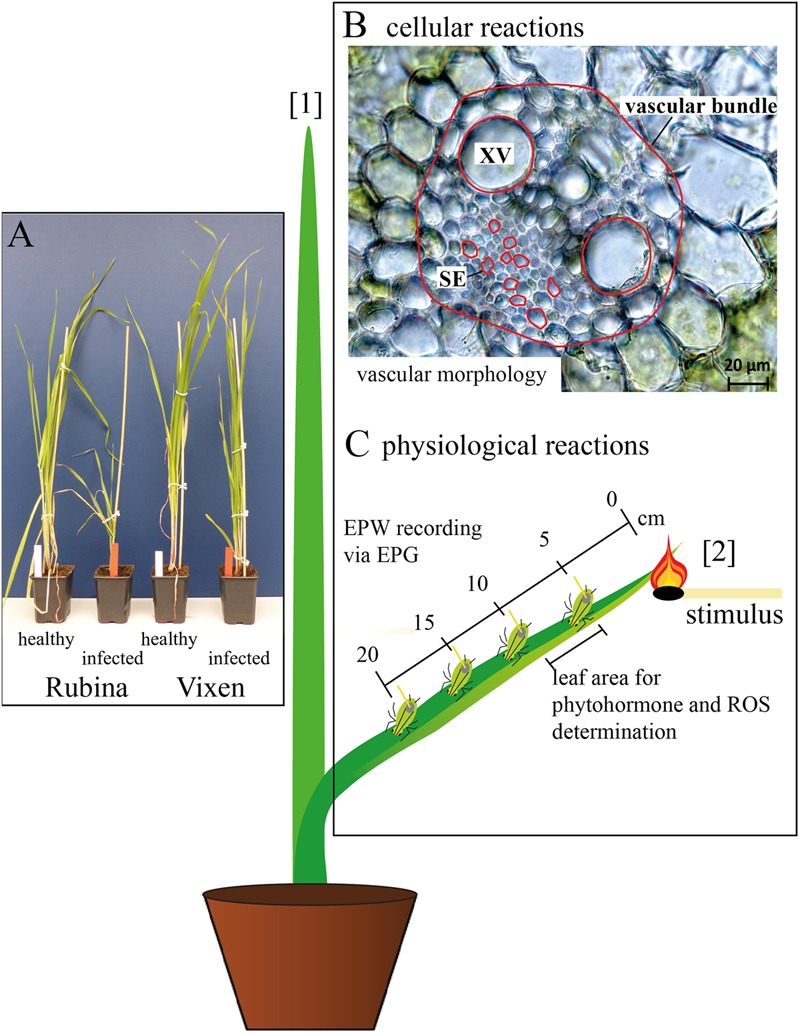
Overview of the experimental set-up. **(A)** The influence of *Barley yellow dwarf virus* (BYDV) infection on *Hordeum vulgare* plants was compared using a susceptible (Rubina) and a tolerant (Vixen) cultivar. **(B)** The pathogenesis was analyzed by determination of the leaf width, vascular bundle area, area of single sieve elements (SE), and area of single xylem vessels (XV) midway from tip to base of the youngest mature leaf (leaf number [2]). The measured areas are outlined in red and leaf numbers shown in brackets. **(C)** The physiological effects on the plant were examined using electrical penetration graph (EPG) recordings from feeding aphids located at different distances (0–20 cm) from a heat stimulus at the tip of leaf number [2]. Additionally, reactive oxygen species (ROS) and defense phytohormones [salicylic acid (SA), abscisic acid, jasmonic acid (JA), JA isoleucine conjugate and JA valine conjugate] were measured for leaf samples extending from 5 to 9 cm from where the heat stimulus was administered.

All experiments were conducted on healthy and BYDV infected plants (below only called infected plants) of the cv. Rubina and the cv. Vixen (Schaller et al., 1964; **Figure 1A**). After four weeks the plants were well infected by BYDV-PAV and used to study the propagation of electrophysiological reactions with the electrical penetration graph (EPG) technique. All other analyses (morphological and chemical parameters) were conducted six weeks after virus infection.

The morphological effects of the infection were solely studied at the middle of the youngest mature leaf (number (no.) [2]) to guarantee the comparability among all considered plants (**Figure 1B**). One leaf per plant was analyzed and considered as a biological replicate. Using a fresh razor blade, one cross section per leaf was made by hand, covered with H_2_O_dest_ and a cover glass and immediately observed with the light microscope. Per cross section two xylem vessels were measured and the mean of both was used for the statistical analyses and creation of graphs. Because of the variability of the sieve elements 10 sieve elements per cross section were measured and their median was used for further analyses and graph preparation.

Another set of plants was used for the measurements of electrophysiological reactions in sieve elements of cv. Rubina and cv. Vixen, where phloem sucking aphids were used as ‘bioelectrodes’ (Furch et al., 2010; Salvador-Recatalà et al., 2014; Zimmermann et al., 2016). The EPG-detected EPWs were recorded in basipetal direction along a single leaf at four distances (5, 10, 15, and 20 cm) and triggered by a heat stimulus at the leaf tip (see **Figure 1C** [2]). The heat stimulus was applied with a lit match for 3–4 s. A replicate was composed of one leaf per plant.

A third set of plants was used to investigate the plant responses with respect to ROS and phytohormone levels. They were investigated 30 min after the heat stimulus (control plants without stimulus) by an analysis of a 4 cm leaf piece 5–9 cm from the leaf tip (leaf no. [2] **Figure 1C**). In rapid succession the plant samples were cut with a fresh razor blade, immediately frozen in liquid nitrogen and afterward ground to a fine powder with the use of mortar and pestle. In order to have enough plant material for the chemical analyses the material of three plants was pooled and considered as one replicate. For ROS analyses the necessary amount of plant tissue was weighed at temperatures of liquid nitrogen and stored at -80°C. For the phytohormone analyses the plant tissue was freeze dried (Alpha 1-4 LD plus; Christ, Osterode am Harz, Germany) prior to weighing.

After execution of the experiments all plants (same leaves as used for the experiments) were controlled for their infection status with the DAS-ELISA test.

### Quantification of Virus Infection by DAS-ELISA

The BYDV content was analyzed after the execution of the experiment from 50 mg leaf material coming from the same leaf investigated in the respective experiment. DAS-ELISA (double antibody sandwich – enzyme-linked immunosorbent assay) was performed according to Clark and Adams (1977) using a polyclonal antiserum (against BYDV-PAV) produced in house at the Julius Kuehn-Institute (JKI). After 1 h of incubation with the enzyme substrate (p-nitrophenyl phosphate), extinction (EXT) was measured at 405 nm with a microtiter plate absorbance reader model Sunrise (Tecan GmbH, Grödig/Salzburg, Austria). As a threshold for a successful positive infection an EXT of >0.4 was used, whereas plants that showed an EXT ≤ 0.04 were considered uninfected (Supplementary Figure 1, tested number of replicates see Supplementary Table 1). Plants which showed EXT between 0.04 and 0.4 were excluded from all analyses because of their indifferent infection state.

### Plant Morphology and Microscopy

Per plant one cross section was done in the middle of the 2^nd^ leaf (midway from tip to base). Each cross section was covered with H_2_O_dest_ and a cover glass and immediately observed with the light microscope (AXIO Imager.M2, Zeiss, Jena, Germany) equipped with a color camera (AXIOCAM 503 color Zeiss, Jena, Germany). Digital images were processed and measurements of the vascular bundle, xylem and phloem areas were conducted with the ZEN software (Zeiss, Jena, Germany). Per cross section two xylem vessels were measured and the mean of both was used for the statistical analyses. Because of the variability of the sieve elements 10 sieve elements per cross section were measured and their median was used for further analyses.

### Electrical Penetration Graph Recording

Randomly selected adult apterous aphids of the species *S. avenae* were prepared for EPG measurements as previously described (Will et al., 2007; Schliephake et al., 2013). Four aphids were simultaneously placed on the lower side of leaf no. [2] and their behavior was observed by continuous EPG recording. EPG recording relevant for the measurement of EPWs was started after at least one aphid had begun sieve tube penetration and ingested phloem sap (Furch et al., 2009). Afterward the leaf tip was carefully burned for 3 s to trigger EPWs. The time of burning was marked by -50 mV calibration signals, triggered by the GIGA-8 EPG amplifier (EPG Systems, Wageningen, Netherlands). Induced reactions were recorded with the GIGA-8 EPG amplifier and EPG stylet software (EPG Systems, Wageningen, Netherlands) for 120 min. Data analysis was conducted using the EPG stylet analysis module in accordance with Tjallingii and Hogen Esch (1993). If an EPW amplitude was detected, the beginning of the amplitude was taken as a basis to calculate the EPW propagation speed. In case the EPW amplitude was masked by aphid behavior associated voltage changes, the observed change of behavior from ingestion (waveform E2) to secretion of watery saliva into sieve tubes (waveform E1) was taken as reference time point for EPW velocity calculation. A transition from ingestion to secretion of watery saliva was described so far in direct association with EPWs in cucurbits (Furch et al., 2010), rapeseed (Zimmermann et al., 2016) and in Arabidopsis (Salvador-Recatalà et al., 2014). Sieve element occlusion might act as a trigger for the observed change in aphid behavior (Will et al., 2007, 2009; Furch et al., 2010). Propagation velocity was calculated for adjacent measuring points by the time of the detected signal and the distance between the two aphids.

Because it is not possible to distinguish between the different electrophysiological reaction types with the EPG technique, i.e., action potential, variation potential and system potential (Felle and Zimmermann, 2007; Zimmermann and Felle, 2009; Zimmermann and Mithöfer, 2013; Zimmermann et al., 2016), we term the recorded electrophysiological reactions EPW (see Furch et al., 2007).

### Analysis of Reactive Oxygen Species With DCFDA

The method employed was adapted from Jambunathan (2010). For extracting ROS from the plant tissue 0.25 mL of 10 mM Tris-HCl buffer (pH 7.2) was added to 20 mg of fresh, ground plant tissue. This mixture was gently shaken at 4°C for 5 min and then centrifuged (12000 × *g*, 20 min, 4°C). The supernatant was transferred into a fresh tube and stored on ice for further usage.

A 10 μL portion of the supernatant was diluted 1:40 in 10 mM Tris-HCl buffer and added in triplicates onto a black 96 well plate (F bottom, chimney well, black Fluotrac; Greiner bio-one, Frickenhausen, Germany). Shortly before measurement, 2,7-dichlorofluorescein diacetate (DCFDA; Sigma–Aldrich, Steinheim, Germany) in dimethyl sulfoxide (DMSO) was added to the plate to achieve a final concentration of 10 μM in a total volume of 200 μL in each well. After gentle agitation and incubation at room temperature in the dark for about 10 min, the emitted fluorescence was measured for 10 s per well with a Tecan multi-well reader (infinite M200, Tecan Austria GmbH) using optimal gain and a total of five flashes. The fluorophore was excited with a wavelength of 485 nm and the emission recorded at 530 nm. The Tecan multi-well reader was operated with the i-control 1.5 software (Tecan Austria GmbH). Hydrogen peroxide (ACROS organics, Thermo Fischer Scientific, Geel, Belgium) was used as an external standard.

### Analysis of Phytohormones

A 1 mL portion of phytohormone extraction buffer [80% MeOH with 0.1% formic acid (FA) spiked with internal standards: 40 ng mL^-1^ D_6_JA (High Purity Compounds (HPC), Cunnersdorf, Germany), D_4_SA (Sigma–Aldrich), D_6_ABA (Santa Cruz Biotechnology, Santa Cruz, CA, United States) and 8 ng mL^-1^ D_6_JA-Ile (HPC)] was added to 10 mg powdered freeze dried plant tissue kept on ice. The samples were mixed for 30 s by vortexing and subsequently sonicated for 15 min at 35 kHz in a water bath at room temperature. A centrifugation step followed (-10°C for 20 min at 4500 × *g*). The supernatant was filtered through a 0.45 mm PTFE AcroPrep^TM^ 96-well filtration plate (Pall Corporation, Port Washington, NY, United States). This filtrate (3 μL) was analyzed by LC-MS/MS via multiple reaction monitoring (MRM) after separating the phytohormones with an Agilent HPLC system on a Zorbax Eclipse XDB-C18 column (50 mm × 4.6 mm, 1.8 μm; Agilent Technologies, Santa Clara, CA, United States). The column temperature was maintained at 25°C and the profile of the mobile phase set as follows: 0.0–0.5 min, 90% of 0.05% (v/v) FA and 10% ACN; 0.5–4.0 min, 10–90% ACN; 4.0–4.5 min, 100% ACN; 4.5–7.0 min, 10% ACN. During the whole separation process the mobile phase was pumped with a flow rate of 1.1 mL min^-1^.

In an API 5000 tandem mass spectrometer (Applied Biosystems, Foster City, CA, United States) MRM was used to analyze parent ion → product ion fragmentation as follows: m/z 136.9 →93.0 [collision energy (CE) -22 V; declustering potential (DP) -35 V] for SA; m/z 140.9 → 97.0 (CE -22 V; DP -35 V) for SA-D_4_; m/z 209.1 → 59 (CE -24 V; DP -35 V) for JA; m/z 215.1/214.1 → 59.0 (CE -24 V; DP -35 V) for JA-D_5/6_; m/z 322.2 → 130.1 (CE -24 V; DP -45 V) for the JA-isoleucine conjugate (JA-Ile); m/z 328.2 → 130.1 (CE -30 V; DP -50 V) for D_6_JA-Ile; m/z 308.19 → 116.1 (CE -30 V; DP -50 V) for the JA-valine conjugate (JA-Val); m/z 263.0 → 153.2 (CE -22 V; DP -35 V) for abscisic acid (ABA); m/z 269.0 → 159.2 (CE -22 V; DP -35 V) for ABA-D_6_. To achieve the mentioned MRMs a Turbospray ion source was operated in negative mode and other parameters maintained as listed below. The ion spray voltage was set to -4500 eV and the turbo gas temperature to 700°C. The nebulizing and heating gasses were adjusted to 60 psi, the curtain gas to 25 psi and the collision gas to 7 psi. The Analyst 1.6 software (Applied Biosystems) was used for data acquisition and analysis. The signals of the internal standards were used to quantify the native phytohormones.

### Statistics

In order to test whether morphological leaf characteristics were dependent on the cultivar and BYDV infection, and whether phytohormone concentrations and ROS production were influenced by infection, burning treatment, and cultivar, factorial ANOVAs followed by Tukey honest significant difference tests were used. The generalized least squares method [gls from the nlme library (Pinheiro et al., 2017)] with the varIdent variance structure was applied in case of variance heterogeneity. Whether the different variance of burning, infection, or cultivar, or a combination of factors should be incorporated into the model, was determined by comparing models with different variance structures with a likelihood ratio test and choosing the model with the smallest Akaike Information Criterion (AIC). The influence (*p*-values) of the explanatory variables was determined by sequential removal of explanatory variables starting from the full model, and comparison of the simpler with the more complex model with a likelihood ratio test (Zuur et al., 2009). Differences between factor levels were determined by factor level reduction (Crawley, 2013). If necessary to achieve normality of the residuals or variance homogeneity, data were transformed as specified in the corresponding tables (**Tables 3, 4**). JA-Val conjugate concentrations were only analyzed for burned plants since this jasmonate conjugate was only sporadically detected in non-burned plants. Morphological, ROS and phytohormone data were analyzed with R version 3.4.1 (R Core Team, 2017).

The presence of electrophysiological reactions at each location was categorized with “yes” and “no” and Fisher’s exact test was used for the comparison of healthy and infected plants for both cultivars. The propagation velocity was compared at each distance for the two treatments and between the barley cultivars by using the non-parametric Steel-Dwass method. Due to the absence of any electrophysiological reactions, data points beyond 5 cm in infected plants of the cv. Rubina were not included in the test for differences of propagations velocity. These data were analyzed with jmp 12 (SAS Institute).

## Results

### The Leaf and Vascular Morphology of the Cultivar Rubina Was Negatively Affected by BYDV

**FIGURE 2:**
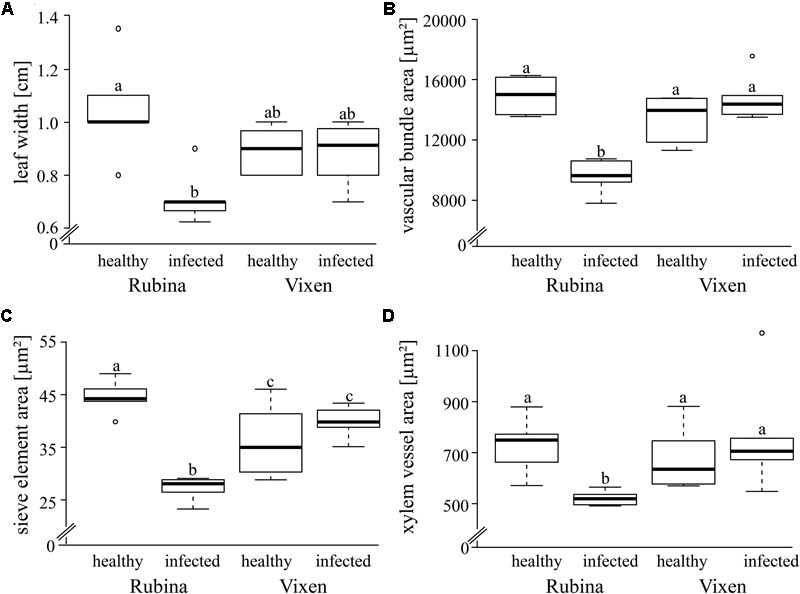
Morphological comparison of healthy and BYDV infected *Hordeum vulgare* plants. The impact of BYDV infection on the morphology of a leaf and the vascular system was examined via various parameters – **(A)** leaf width (cm), **(B)** vascular bundle area (μm^2^), **(C)** sieve element area (μm^2^) and **(D)** xylem vessels area (μm^2^). The study included a susceptible (Rubina) and a tolerant (Vixen) cultivar. The bold horizontal line in the box illustrates the median value, boxes present the interquartile range. Note the suppressed zero at the *y*-axis scale! Different letters indicate statistical differences (*p* = 0.05; *N* = 5–6).

Since BYDV is localized in the phloem, it can be assumed to have a direct effect on this tissue. We found that the infection of the cv. Rubina significantly decreased the leaf width (-31%), vascular bundle area (-35%), sieve element area (-39%) and xylem vessel area (-29%) compared to that of uninfected plants. However, the tolerant cv. Vixen was not affected by infection (**Figure 2**, statistics see **Table 1**).

**Table 1:** Statistics of the analysis of morphological leaf traits of the two *Hordeum vulgare* cultivars Rubina (susceptible) and Vixen (tolerant) after BYDV infection.

Morphological trait	Statistical test used	Variance structure	Factor	*F*/*L*-ratio	*P*-value
Leaf width	ANOVA		Cultivar	0.001	0.978
			Infection	9.091	**0.007**
			Interaction	8.640	**0.008**
Vascular bundle area	ANOVA		Cultivar	8.476	**0.009**
			Infection	9.867	**0.005**
			Interaction	34.115	**<0.001**
Sieve element area	GLS	varIdent(form = ∼1|infection)	Cultivar	*8.443*	**0.004**
			Infection	*4.682*	**0.030**
			Interaction	*21.235*	**<0.001**
Xylem area	GLS	varIdent(form = ∼1|combi)	Cultivar	<*0.001*	0.386
			Infection	*13.857*	**<0.001**
			Interaction	*6.352*	**0.012**

*Significant P-values (≤0.05) are given in bold. Depending on which statistical test was used, F-values or Likelihood ratios (L-ratio) are given. Likelihood ratios are given in italics. Interaction means the statistical interaction between cultivar and infection. Combi in the variance structure means the combination of all two main factors (cultivar, BYDV infection) i.e., each box in the boxplot of **Figure 2D** was allowed to have its own variability (N = 5–6).*

### The Electrophysiological Conductivity of the Phloem Was Impaired by BYDV Infection

The propagation of electrophysiological reactions in plants is believed to take place largely in phloem tissue (Zimmermann and Mithöfer, 2013; Hedrich et al., 2016). Since electrophysiological propagation requires coupling of the individual sieve elements, BYDV colonization of these elements may impair electrophysiological conductivity. This hypothesis was tested with EPG measurements following application of a heat stimulus (**Figure 1**).

**FIGURE 3:**
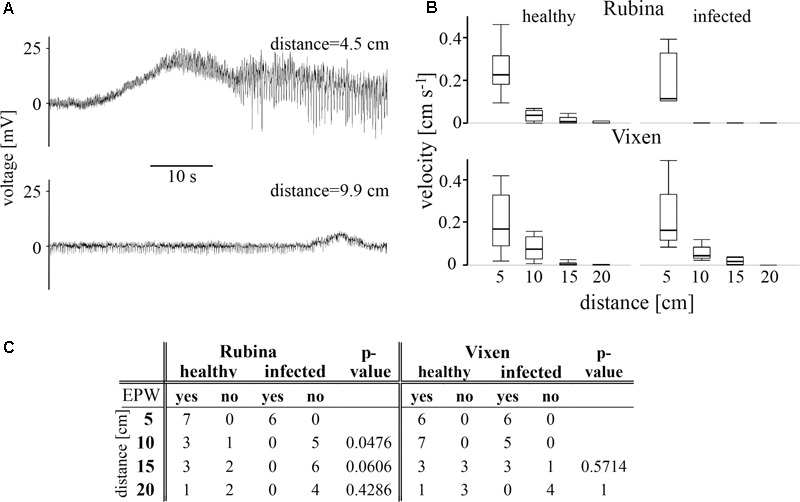
Measurement of electropotential waves (EPW) using aphids as bioelectrodes. EPWs were detected with an EPG approach. **(A)** Two typical recordings of heat-triggered EPWs showing a decline in amplitude and duration with increasing distance from the stimulus site in a healthy plant of the cultivar Vixen. **(B)** The velocities of EPWs in healthy and BYDV infected plants of both cultivars (the susceptible Rubina and the tolerant Vixen) are calculated for each distance (5–20 cm). The bold, horizontal line illustrates the median value. The propagation velocity was compared at different distances from the stimulus site for the two treatments and between the barley cultivars by using the non-parametric Steel-Dwass method. **(C)** The presence of EPWs after each heat stimulation at different distances from the stimulus site is summarized. Healthy and infected plants at a certain distance were compared for Rubina and Vixen separately with the Fisher’s exact test (*N* = 8–11).

The results showed that the heat stimulus triggered an unspecific electrophysiological reaction henceforward called EPW (see also Furch et al., 2007), which exhibited a decreasing velocity with increasing distance, although there is mostly no statistically significant difference between adjacent measuring points within one treatment (**Figure 3A**, statistical values see **Table 2**). Independent of the cultivar and the infection status we observed EPWs 5 cm downstream of the stimulus site (**Figures 3B,C** and Supplementary Figure 2). However, in the infected cv. Rubina no EPW was detected at distances from 10 to 20 cm (**Figure 3C**). In healthy plants of this cultivar the detection rate of EPWs decreased with distance from the site of burning, as well, but EPWs were recorded as far as 20 cm from the leaf tip. In the cv. Vixen, the detection rate of EPWs was comparable in infected and healthy plants at the respective distances (**Figure 3C**).

**Table 2:** Statistical values for the analysis of EPG recording based signal velocity between adjacent measuring points of one treatment and between respective measuring points of two *Hordeum vulgare* cultivars according to BYDV infection.

Morphological trait	Statistical test used	Distance to leaf tip [cm]	*N*	*P*-value treatment	*P*-value healthy/inf
Rubina	Steel-Dwass	5	7	0.052	0.861
		10	4	0.819	n.t.
		15	5	n.t.	n.t.
		20	3	-	n.t.
Rubina infected	Steel-Dwass	5	6	**0.028**	
		10	5	n.t.	
		15	6	n.t.	
		20	4	-	
Vixen	Steel-Dwass	5	6	0.438	1.000
		10	7	**0.026**	0.916
		15	6	0.889	0.690
		20	4	-	0.877
Vixen infected	Steel-Dwass	5	6	0.066	
		10	5	0.256	
		15	4	0.264	
		20	4	-	

*Measuring points with N < 4 or where no signal was recorded were not included in a statistical comparison (n.t.). Significant P-values are given in bold.*

The velocity of the EPWs also diminished in healthy and infected plants of both cultivars with increasing distance from the stimulus site but mostly no statistical difference was observed between adjacent measuring points (**Figure 3B** and **Table 2**).

### The ROS Formation Was Dependent on Infection Status and Burning Treatment

**FIGURE 4:**
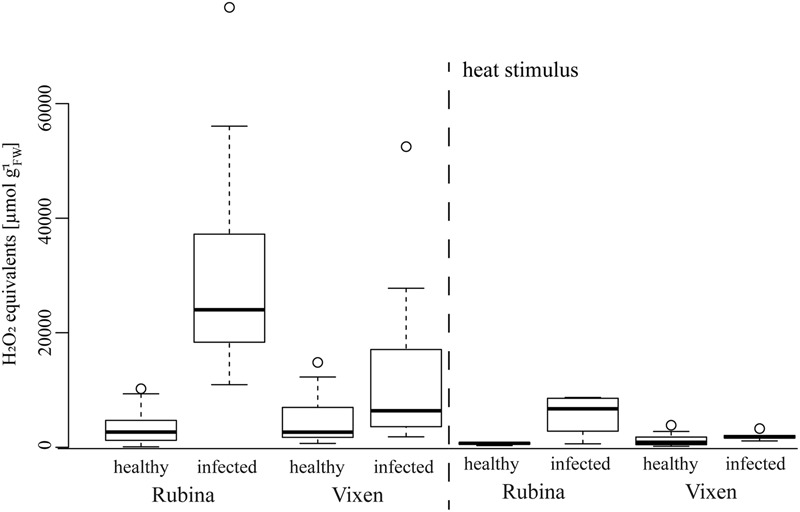
Reactive oxygen species distribution in healthy and BYDV infected *H. vulgare* cultivars Rubina (susceptible) and Vixen (tolerant) with and without heat stimulus. The general defense response of BYDV susceptible (Rubina) and tolerant (Vixen) cultivars was examined by the measurement of the H_2_O_2_ concentrations after BYDV infection and 30 min after a heat stimulus at the leaf tip. Infected Rubina plants showed a higher concentration of ROS than uninfected ones. The bold horizontal line in the box illustrates the median value, boxes represent the interquartile range. *N* = 4–19, exact number of replicates see Supplementary Table 2.

The negative effect of the infection on the phloem especially in the cv. Rubina (**Figures 2, 3**) raised questions about the underlying factors. One possible factor might be the general physiological stress level induced by BYDV, which might be reflected in the level of ROS. For both cultivars, we found low ROS levels in healthy plants. However, after infection ROS levels were significantly higher in plants of the cv. Rubina compared to plants of the cv. Vixen (**Figure 4** and **Table 3**). To evaluate the magnitude of the influence of BYDV infection on the ROS formation during early signaling events in response to abiotic stresses, we applied an additional heat stimulus. Following the heat stimulus, we observed an equal significant decrease of ROS formation in a remote area (*d* = 5–9 cm) for both cv. Rubina and cv. Vixen (**Figure 4** and **Table 3**).

**Table 3:** Statistics of the analysis of ROS of the two *Hordeum vulgare* cultivars Rubina (susceptible) and Vixen (tolerant) after BYDV infection and heat stimulus.

Statistical test used	Transformation/variance structure	Factor	Likelihood-ratio	*P*-value
gls	log	Cultivar	1.470	0.225
	varIdent(form = ~1|combi)	Infection	17.105	**<0.001**
		Heat	44.582	**<0.001**
		Cultivar:infection	18.209	**<0.001**
		Cultivar:heat	0.044	0.834
		Infection:heat	0.384	0.535
		Cultivar:infection:heat	0.620	0.431

*Significant P-values are given in bold. Combi in the variance structure means the combination of all three main factors (cultivar, BYDV infection, heat stimulus) i.e., each box in the boxplot of **Figure 4** was allowed to have its own variability. N = 4–19, exact number of replicates see Supplementary Table 2.*

### ABA and SA Were Differentially Influenced Depending on BYDV Infection and Heat Stimulus

**FIGURE 5:**
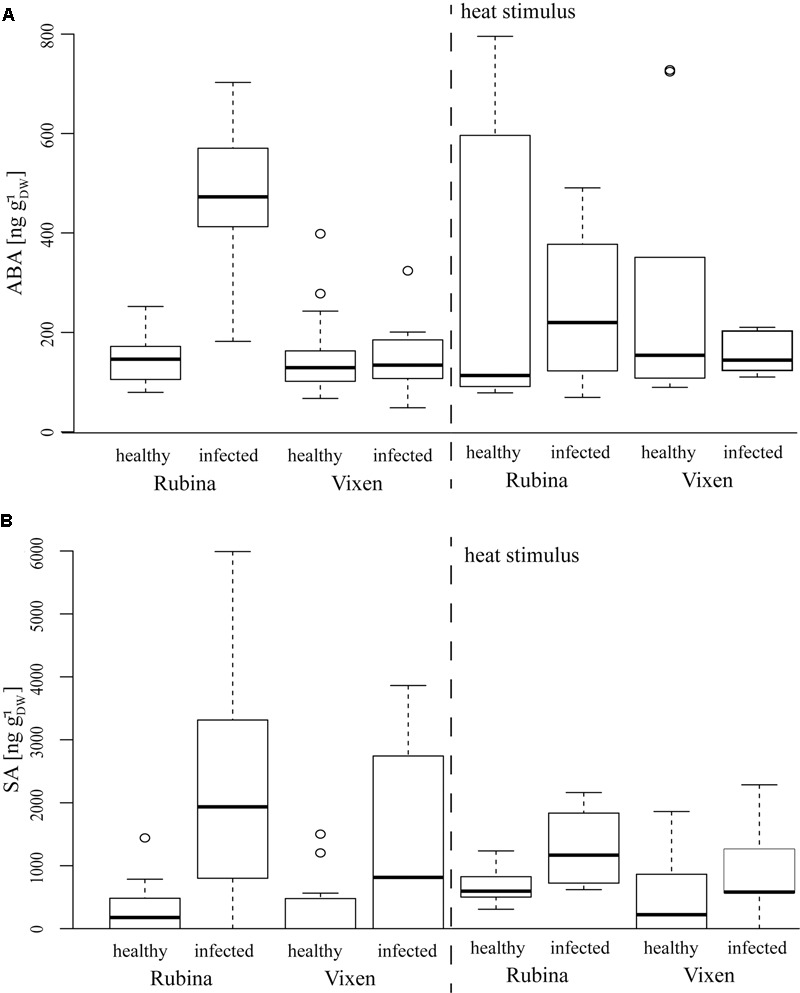
Phytohormone concentrations after BYDV infection and heat stimulus in the *H. vulgare* cultivars Rubina (susceptible) and Vixen (tolerant). **(A)** Absicic acid (ABA) levels are increased in BYDV infected plants, whereas **(B)** SA is increased in infected plants of both cultivars. Burning does not differentially influence the phytohormone levels in the different treatments. The bold horizontal line in the box illustrates the median value, boxes present the interquartile range. *N* = 4–18, exact number of replicates see Supplementary Table 2.

An additional indicator of a general physiological stress is the level of the phytohormone ABA which is also associated with ROS (Gilroy et al., 2016). The ABA concentrations in healthy cv. Rubina and cv. Vixen plants were similar. Akin to the ROS formation only the cv. Rubina showed increased ABA levels after infection. The heat stimulus did not influence ABA concentrations in either cultivar, regardless of the infection status (**Figure 5** and **Table 4**).

**Table 4:** Statistical values for the analysis of phytohormone concentrations in the two *Hordeum vulgare* cultivars Rubina (susceptible) and Vixen (tolerant) according to BYDV infection and heat stimulus.

Phytohormone	Statistical test used	Transformation/variance structure	Factor	*F*/*L-ratio*	*P*-value
ABA	GLS	log	Cultivar	*0.959*	0.327
		varIdent(form = ~1|combi)	Infection	*0.616*	0.433
			Heat	*2.100*	0.147
			Cultivar:infection	*25.053*	**< 0.001**
			Cultivar:heat	*0.834*	0.361
			Infection:heat	*2.710*	0.100
			Cultivar:infection:heat	*1.740*	0.187
SA	ANOVA	square root	Cultivar	3.950	**0.050**
			Infection	25.634	**< 0.001**
			Heat	0.823	0.367
			Cultivar:infection	0.953	0.332
			Cultivar:heat	0.109	0.743
			Infection:heat	1.939	0.168
			Cultivar:infection:heat	0.518	0.474
JA	GLS	square root	Cultivar	*0.196*	0.658
		varIdent(form = ~1|combi)	Infection	*4.032*	**0.045**
			Heat	*50.260*	**< 0.001**
			Cultivar:infection	*6.894*	**0.009**
			Cultivar:heat	*2.995*	0.083
			Infection:heat	*1.016*	0.313
			Cultivar:infection:heat	*7.542*	**0.006**
JA-Ile	GLS	square root	Cultivar	*2.203*	0.138
		varIdent(form = ~1|combi)	Infection	*4.833*	**0.028**
			Heat	*62.306*	**< 0.001**
			Cultivar:infection	*5.687*	**0.017**
			Cultivar:heat	*2.531*	0.112
			Infection:heat	*2.067*	0.150
			Cultivar:infection:heat	*13.375*	**< 0.001**
JA-Val	ANOVA	log	Cultivar	0.086	0.772
			Infection	10.665	**0.004**
			Cultivar:infection	2.943	0.101

**FIGURE 6:**
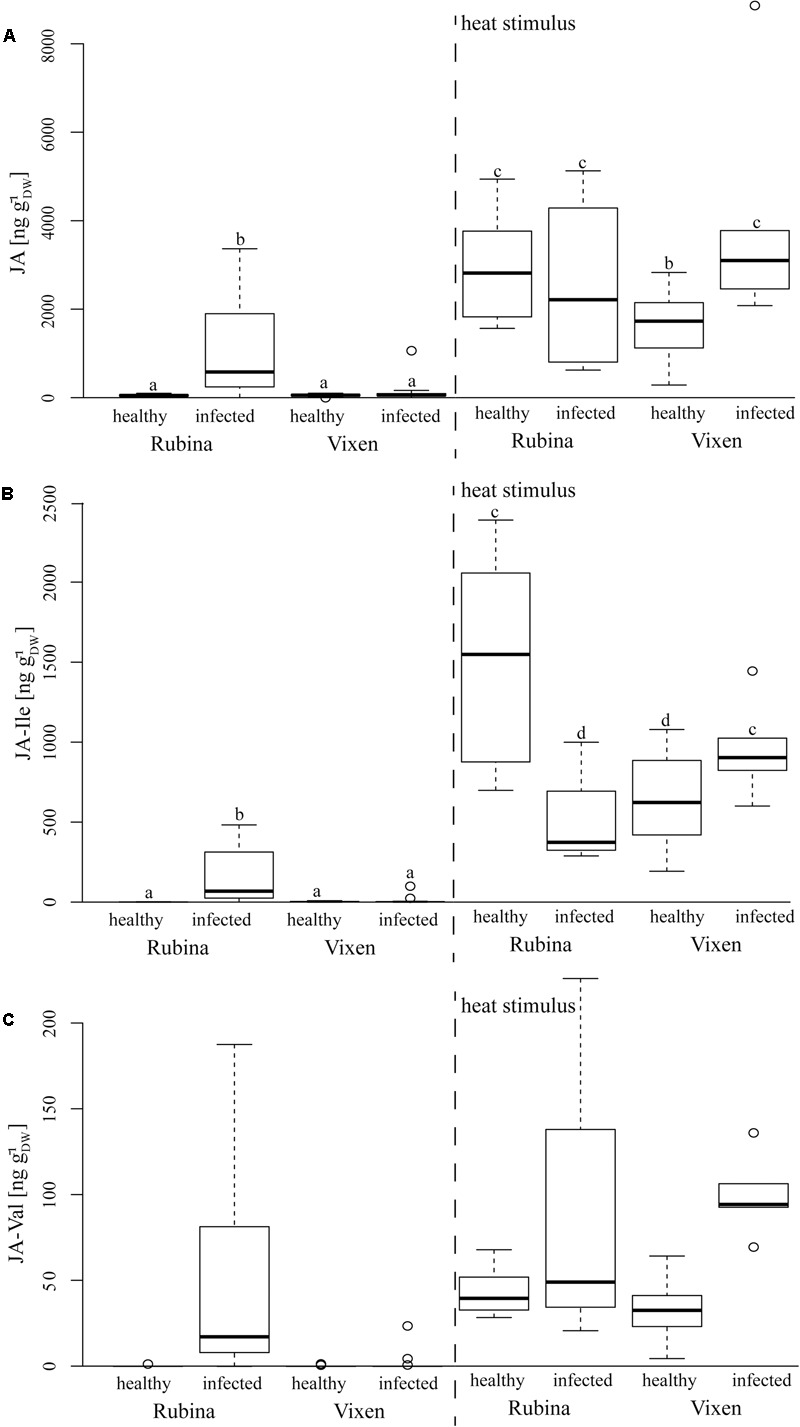
Concentrations of several jasmonates in response to BYDV infection and a heat stimulus in the *Hordeum vulgare* cultivars Rubina (susceptible) and Vixen (tolerant). The jasmonates – **(A)** JA, **(B)** jasmonic acid isoleucine conjugate (JA-Ile), and **(C)** jasmonic acid valine conjugate (JA-Val) – were only detected in reaction to BYDV infection and a heat stimulus at the leaf tip. The bold horizontal line in the box illustrates the median value, boxes present the interquartile range. Different letters indicate statistical differences (*p* < 0.05; *N* = 4–18, exact number of replicates see Supplementary Table 2).

*Significant P-values are given in bold. Depending on which statistical test was used F-values or Likelihood ratios (L-ratio) are given. Likelihood ratios are given in italics. Interaction means the statistical interaction between cultivar and infection. Combi in the variance structure means the combination of all three main factors (cultivar, BYDV infection, heat stimulus) i.e., each box in the boxplot of **Figures 5, 6** was allowed to have its own variability. JA-Val conjugate concentrations were only analyzed for burned plants since this jasmonate conjugate was only sporadically detected in non-burned plants. N = 4–18, exact number of replicates see Supplementary Table 2.*

Salicylic acid concentrations were in general slightly higher in plants of the cv. Rubina compared to plants of the cv. Vixen (**Figure 5** and **Table 4**). Whereas infection led to increased SA concentrations in both cultivars, burning did not significantly change the SA concentrations in either cultivar (**Figure 5** and **Table 4**).

### Jasmonates Were Increased After BYDV Infection

The JA pathway was also investigated to study the stress reaction to BYDV and heat stimuli. Three different jasmonates – JA, the JA-Ile and the JA-Val conjugate – were studied as representatives of the JA pathway. Similar to ABA concentrations, we found that JA, JA-Ile, and JA-Val concentrations were significantly increased in non-heat stimulated but infected cv. Rubina plants but not in infected cv. Vixen plants (**Figure 5** and **Table 4**). The jasmonate concentrations in non-heat stimulated cv. Vixen plants were either very low or not detectable (JA-Val) regardless of the infection status.

In contrast to infection, the heat stimulus led to a marked increase in jasmonate concentrations in healthy and infected plants of both cultivars (**Figure 5** and **Table 4**). However, whilst infected cv. Vixen plants showed higher jasmonate concentrations upon burning than uninfected cv. Vixen plants, infected cv. Rubina plants showed either higher (JA- Val), similar (JA) or even lower concentrations (JA-Ile) than uninfected plants. The general rise of jasmonate concentrations due to the heat stimulus was significantly stronger than that due to BYDV infection thereby showing that the maximum possible jasmonate concentration was not reached during infection.

## Discussion

Susceptible barley plants like the cv. Rubina show strong symptoms such as stunted growth and yellow or red discoloration of the leaf tips whereas tolerant cultivars like the cv. Vixen with a *Ryd2* based tolerance (Schaller et al., 1964) show no or only weak symptoms.

*Barley yellow dwarf virus* is localized in the phloem and is persistently transmitted by aphids. Due to its phloem location, the virus negatively affects the integrity of the sieve elements in susceptible barley cultivars (Esau, 1957). However, despite extensive study (Schaller et al., 1964; Niks et al., 2004; Scholz et al., 2009; Schliephake et al., 2013) the effect of BYDV on phloem parameters and plant defense responses, including changes in ROS and defense hormones, have to our knowledge not been reported.

### Phloem Infection Leads to Decreases in Vascular Development and Reduced Propagation of Electropotential Waves

With regard to development, vascular bundles develop in healthy and BYDV infected leaves in a comparable way and there is no influence on the fundamental organization of the phloem tissue (Esau, 1957). Our observations show that the phloem as well as the xylem area were both negatively affected due to BYDV infection, which could be explained by necrotic obliteration of sieve elements and xylem vessels, already occurring in differentiating vascular bundles in highly susceptible barley (Esau, 1957). Pathological degeneration and necrosis of the phloem, involving sieve elements, CCs and parenchyma cells, affects old and young vascular bundles in main and marginal veins.

*Barley yellow dwarf virus* infection affects the physiological functionality of sieve elements with regard to their propagation of EPWs as indicated by our results obtained by using aphids as bio-electrodes. EPWs show a decreasing propagation velocity along sieve tubes with increasing distance from the trigger site as previously shown in cucurbits and rapeseed (Furch et al., 2010; Zimmermann et al., 2016) and observed here for healthy plants of both cultivars and BYDV infected tolerant plants. A decrease in electrophysiological conductivity of the phloem in the BYDV infected susceptible cultivar is already indicated because of the reduced radius of single sieve elements as well as the reduced total radius of vascular bundles, combined with necrosis of single sieve elements (Esau, 1957). Moreover, there is likely to be an increase in the longitudinal (intracellular) resistance for electrophysiological signals according to the cable theory model for the calculation of the electrophysiological current along passive neurites (Taylor, 2013) and sieve tubes in plants (Hedrich et al., 2016) showing that a reduced cell radius will increase the longitudinal (intracellular) resistance for current flow. As a further effect, the lateral resistance (affecting the flow of electrophysiological current through the membrane) will decrease with decreasing cell diameter due to a relative increase of the membrane surface with regard to the cell volume. Together both parameters would negatively affect the propagation of electrophysiological current along a cable, leading to the observed loss of EPWs near to their trigger site. It can be suggested that the suppression of EPW propagation may have a negative effect on, e.g., plant defense (Trêbacz et al., 2006).

The cable theory is used to explain the propagation of action potentials along neurites and was recently used for the description of electrophysiological signals in sieve tubes as mentioned above (Hedrich et al., 2016). While a neurite is part of a single cell, sieve tubes are composed out of several sieve elements, each of it a single cell with individual electrophysiological properties. Latter appears to be a strong difficulty in simply transferring the theory to the plant system where it does not reflect the complexity of the biological systems. Due to lacking basal experimental studies about the transfer of electrophysiological potentials in sieve tubes it is not possible to use the cable theory to fully explain our observations. A parameter that appears to be relevant for such calculations is the sieve element and vascular bundle size (area, length) that cannot be determined without great effort when using aphids as bio-electrodes. Aphids at the different measuring points could feed from the same sieve tube or from different vascular bundles, which might have effects on the amplitude and velocity of an EPW.

Further factors that might have an effect on the lateral and longitudinal resistance is (i) the connection to sieve elements with CCs via pore plasmodesm units (PPUs) and (ii) the connection of adjacent sieve elements via sieve pores. Plant viruses are able to modulate the opening state of plasmodesmata by using so called movement proteins (Deom et al., 1992) to help them spread (Lazarowitz and Beachy, 1999). ORF4 of BYDV encodes a 17 kDa called movement protein (Nass et al., 1998) that is required for systemic infection of the virus in barley (Chay et al., 1996). We suggest that the BYDV movement protein triggers opening of PPUs and sieve pores. The opening state of plasmodesmata is in addition also affected by plant signaling and defense compounds whereas ROS and SA trigger plasmodesmata closure (Cheval and Faulkner, 2017). Since systemic virus infection occurs in both barley cultivars tested, we suggest that PPUs and sieve pores are opened in both cultivars studied. If closure of both plasmodesmata types is not triggered by plant defense compounds, although a significant increase of ROS was caused by BYDV infection in the two cultivars can be suggested because of systemic BYDV infection. However, this remains only speculation and has to be tested in later experiments.

### Viral Infection Induces a Greater Response of Chemical Signals in the Susceptible Than the Tolerant Cultivar

One of the reactions of plants to invasion of pathogens including viruses is the generation of ROS (Bolwell and Wojtaszek, 1997). In response to infection, we observed an increase of ROS (measured as H_2_O_2_ equivalents; **Figure 4**), mainly in the susceptible cultivar similar to ROS accumulations previously reported after infection by the *Plum pox virus* (Hernandez et al., 2006; Diaz-Vivancos et al., 2008), *Cucumber mosaic virus* (Riedle-Bauer, 2000) and *Clover mosaic virus* (Clarke et al., 2002). Thus, increased ROS production seems to be a common defense response against viral infection.

We observed a significant increase of ABA concentrations in infected cv. Rubina plants. ABA accumulation promotes closing of stomata and consequently the reduction of transpiration and, therefore, water loss. Another consequence is the decrease in gas exchange leading to the reduction of the photosynthetic activity (Cutler et al., 2010; Brandt et al., 2012; Mittler and Blumwald, 2015). The result is a reduction in organ size, vascular bundle development and growth of the whole plant, as it is shown here for infected cv. Rubina plants (**Figures 1A, 2**) and an early senescence (Wehner et al., 2015). The stunted growth of infected cv. Rubina plants (**Figures 1A, 2**) can thus be attributed to a shortage of photoassimilates due to the increased levels of both ROS (**Figure 4**) and ABA (**Figure 5**). Further investigation of stomatal guard cells might help to evaluate this hypothesis.

Reactive oxygen species signaling in response to different stresses is also known to interfere with SA signaling (Herrera-Vasquez et al., 2015). Both infected barley cultivars showed an increase in SA concentrations compared to healthy plants, whereas the heat stimulus did not induce a response (**Figure 5B**). Davis et al. (2015) showed a similar increase in SA for BYDV infected wheat plants. SA promotes ROS production, on the one hand, and ROS scavenging, on the other, in a temporally dynamic way (Herrera-Vasquez et al., 2015). The increase in both ROS and SA in susceptible plants upon virus infection suggests a concerted action of these signaling compounds. Increased SA concentrations have also been implicated in reduced growth due to an influence on the lignin content (Gallego-Giraldo et al., 2011) giving an alternative explanation for the reduced growth of infected cv. Rubina.

Infection of plants by pathogens often results in changes in the level of various phytohormones, such as SA and JA (Robert-Seilaniantz et al., 2007). Although, SA and JA signaling pathways are mutually antagonistic, evidences of synergistic interactions have also been reported (Mur et al., 2006). In natural environments when plants cope with multiple attackers as well as abiotic stresses, complex responses are observed (Bari and Jones, 2009), and so it is not yet known how plants prioritize one response over the other. We found marked increases of JA, JA-Ile, and JA-Val concentrations in infected cv. Rubina plants indicating the activation of JA signaling networks in this cultivar (**Figure 6**). These jasmonates also increase upon burning in all treatments keeping with previous reports (Herde et al., 1996), however, this is the first time that JA-Val, has been implicated to be involved in this response as well. In contrast, the cv. Vixen did not display a significant increase in jasmonate levels upon viral infection. This implies a difference in this branch of the defense pathway. One may speculate that the lack of activation of the JA pathway is involved in tolerance of BYDV by Vixen. Low JA levels do not inhibit SA signaling, which may directly target anti-viral defenses. On the other hand, low jasmonate levels after infection may also be a consequence of viral tolerance if another mechanism antagonizes viral infection and so defense signaling is not induced in comparison to the susceptible cultivar.

## Conclusion

The susceptible barley cultivar Rubina and the tolerant cultivar Vixen were found to differ markedly after BYDV infection in various aspects of their phloem anatomy, electrophysiological reactions and chemical defense signaling. The reduced growth of the susceptible cultivar appears to result from increased ABA and ROS level and reductions in vascular development and suppressed long distance electrophysiological reactions. The susceptible cultivar also displayed elevated concentrations of the defense hormones ABA, SA, and jasmonates after viral infection. The superior performance of the tolerant cultivar, which carries the *Ryd2* gene, was found to be associated with low levels of hormone signaling, providing new markers for tolerance and a new context for investigating the basis of viral tolerance in barley and other plant species (Ordon et al., 2009).

## Author Contributions

MKP, GK, MRZ, ACUF, and TW designed the research. MKP, GK, NT, AL, DM, ACUF, and TW performed the research. MKP, GK, MRZ, RO, JG, ACUF, and TW analyzed the data. MKP, GK, MRZ, AH, FO, ACUF, and TW wrote the paper.

## Conflict of Interest Statement

The authors declare that the research was conducted in the absence of any commercial or financial relationships that could be construed as a potential conflict of interest.
