# Supplementary material for: Barley yellow dwarf virus Infection Leads to Higher Chemical Defense Signals and Lower Electrophysiological Reactions in Susceptible Compared to Tolerant Barley Genotypes
Source: Front Plant Sci. 2018 Mar 6;9:145. doi: 10.3389/fpls.2018.00145 (PMC5845851; doi:10.3389/fpls.2018.00145)
Supplement: Supplementary file 1 [file Data_Sheet_1.DOCX]

**Suppl. Figure 1: Infection levels between infected Rubina and Vixen plants.** A) Infection status for the tissues used for studies of morphology were compared with a Welch two sample t-test. The infection levels of infected Rubina and Vixen plants were not different (t = 0.844, p = 0.423, N=5-6, exact number of replicates see suppl. table 1) B) Levels for reactive oxygen species and phytohormones were compared with a Wilcoxon rank sum test. The infection levels of infected Rubina were slightly higher than that of Vixen plants (W = 245.500, p = 0.051, N=17-31, exact number of replicates see suppl. table 1). C) Levels for electrical penetration graph were compared with a Welch two sample t-test. The infection levels of infected Rubina and Vixen plants were not different (t = 1.877, p = 0.091, N=8-11, exact number of replicates see suppl. table 1).

**Suppl. Table 1: Number of replicates tested for their infection levels**

| **experiment** | **Rubina healthy** | **Rubina infected** | **Vixen healthy** | **Vixen infected** |
| --- | --- | --- | --- | --- |
| morphology | 6 | 5 | 6 | 6 |
| ROS and hormones | 27 | 17 | 31 | 21 |
| EPG | 9 | 9 | 11 | 8 |

**Suppl. Table 2: Number of replicates used for ROS and phytohormone determination**

| **experiment** | **control** | | | | **with heat stimulus** | | | |
| --- | --- | --- | --- | --- | --- | --- | --- | --- |
|  | **Rubina** | | **Vixen** | | **Rubina** | | **Vixen** | |
|  | **healthy** | **infected** | **healthy** | **infected** | **healthy** | **infected** | **healthy** | **infected** |
| ROS | 19 | 11 | 19 | 16 | 8 | 4 | 12 | 5 |
| hormones | 18 | 12 | 18 | 14 | 6 | 4 | 10 | 5 |

**Suppl. Figure 2: Electropotential waves (EPW) and the transition of aphid behaviour.**

Electrical penetration graph measuring tracks (A-D) show EPWs (measured at the first measuring point 5 cm away from the leaf tip) that are accompanied by a transition of behaviour from ingestion (E2) to the secretion of watery saliva (E1). This change of behaviour, occurs in healthy susceptible (Rubina (A)) and tolerant (Vixen (C)) plants as well as in infected (Rubina (B), Vixen (D)) and is used as an indicator for the transition of an EPW in case that a depolarisation was not detected due to masking by aphid induced resistance and voltage changes. Where an EPG measuring track shows an EPW depolarisation phase it is highlighted in grey.
