# Supplementary figures and images for: Barley yellow dwarf virus Infection Leads to Higher Chemical Defense Signals and Lower Electrophysiological Reactions in Susceptible Compared to Tolerant Barley Genotypes
Source: Front Plant Sci. 2018 Mar 6;9:145. doi: 10.3389/fpls.2018.00145 (PMC5845851; doi:10.3389/fpls.2018.00145)

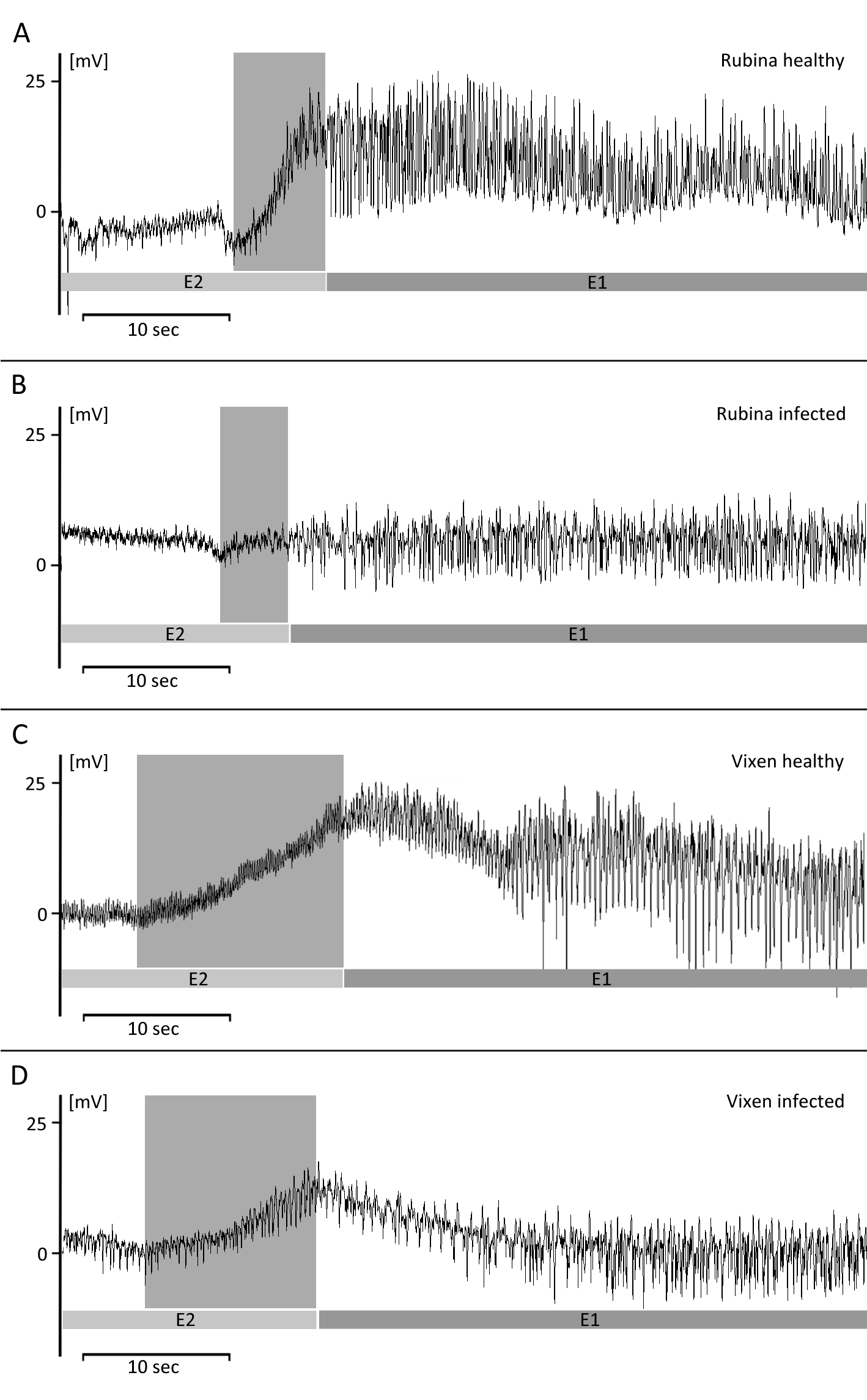

Supplement: Supplementary file 3 [file Image_2.TIF]
